# Development of the patient approach and views toward healthcare communication (PAV-COM) measure among older adults

**DOI:** 10.1186/1472-6963-12-289

**Published:** 2012-08-30

**Authors:** Derjung M Tarn, Henry N Young, Benjamin M Craig

**Affiliations:** 1 Department of Family Medicine, David Geffen School of Medicine, University of California, Los Angeles, USA; 2 School of Pharmacy, University of Wisconsin Madison, Madison, USA; 3 Health Outcomes & Behavior, Moffitt Cancer Center, University of South Florida, Tampa, USA

**Keywords:** Physician-patient communication, Patient activation, Factor analysis

## Abstract

**Background:**

This study examines the psychometric properties of 9 items on the Patient Activation component of the Medicare Current Beneficiary Survey (MCBS) that assess how patients approach and communicate with their physicians. The MCBS is a nationally representative, cross-sectional survey of Medicare beneficiaries.

**Methods:**

We analyzed MCBS data collected in 2002 and 2005 from 15,165 adults aged 65 and older. Exploratory factor analysis was conducted using maximum likelihood to estimate a polychoric correlation matrix on the 2002 data, and confirmatory factor analysis was performed using the 2005 data.

**Results:**

Exploratory factor analysis of the 2002 data showed a 2-factor solution: approach to interactions (5 items) and views about physician’s healthcare communication (6 items). Findings were confirmed using the 2005 data. Items were combined to form the Patient Approach and Views toward Healthcare Communication (PAV-COM) scale (range 1 to 100; Cronbach’s alpha of 0.75, and item-rest correlations between 0.33 and 0.54). Higher PAV-COM scores were associated with greater fulfillment of preventive health behaviors such as vaccinations and cancer screenings.

**Conclusions:**

The PAV-COM measure is a valid tool for assessing patient approaches and views toward communication with physicians. This measure can be used to evaluate interventions to improve patient participation during healthcare encounters.

## Background

Good physician-patient communication results in better patient outcomes [1]. Patients given more information during an office visit are more satisfied, recall more information, and are more adherent to treatment plans [2-4]. Patients have substantial influence on what is discussed. Those who ask more questions typically receive more answers from their physicians [5-7], and those who are more involved in their office visits adhere better to treatment recommendations [4]. Patients who actively seek information and participate more with their physicians report better health status at follow-up [8], have fewer functional limitations, and better health outcomes [9,10].

Despite the importance of patient participation during the medical encounter, most existing survey instruments do not measure how actively patients prepare for or view communication with their physicians. Analyses of audio and video recordings of encounters can provide information about what actually occurs during office visits [11,12], but these analyses are difficult to collect in larger studies. They also do not capture patient understanding or views about the information conveyed during encounters. Survey instruments measuring patient activation fail to capture patient perceptions about communication with their physicians. The primary instruments used to measure patient activation focus on patients’: 1) beliefs about the importance of being activated, 2) confidence and knowledge to act upon those beliefs, and 3) ability to implement and maintain desired actions [13,14]. Patient-centered care measures provide assessments of physician communication, but they mostly assess interactions during specific office visits, and are therefore limited for exploring more general patient views about a healthcare provider [15].

There is a need for an instrument to measure both how actively patients approach and view communication during their healthcare encounters, because these interactions can help shape patient understandings about disease processes and their requisite treatments, and contribute to the development of acceptable treatment plans. This study investigates the psychometric properties of 9 items assessing how actively patients approach and view communication with their healthcare providers. These questions were asked in the 2002 and 2005 versions of the Medicare Current Beneficiary Survey (MCBS), a nationally representative, cross-sectional survey of Medicare beneficiaries. It contains a set of questions assessing patient information-seeking during and outside of interactions with physicians, and patient impressions of interactions with their physicians. This study will investigate the psychometric properties of these MCBS survey items using factor analysis.

## Methods

### Study sample

The MCBS has been administered since 1991. Data used for this study are from the 2002 and 2005 summer and fall supplements of the Access to Care components. Responses were collected via face-to-face interviews from all MCBS respondents who were not institutionalized and did not require a proxy during the interview. The survey response rate was 82.6% [16]. We examined respondents aged 65 or older (N = 7,839 in the 2002 survey and 7,433 in the 2005 survey) because it is unknown whether younger Medicare beneficiaries, who may have more serious illnesses, interact differently with their providers. Prior to analysis, we removed 64 respondents (0.4%) due to non-response and 43 respondents (0.3%) who reported not knowing the answers to 5 or more survey items. The study was approved by the University of Wisconsin’s institutional review board (45 CFR 46.101(b)(4)).

### Survey instruments

The summer supplement of the 2002 and 2005 MCBS surveys both contained 16 questions that were “designed to assess the degree to which Medicare beneficiaries actively participate in their own health care and the decisions concerning that health care.” [17] Our analyses focused on 9 of the 16 items. These items queried patients about their preparation for healthcare encounters and about their views concerning communication with their healthcare providers. We did not analyze 7 items related to patient self-efficacy (confidence in performing specified activities) or self-care.

The survey questions are listed in Table 1. All had 4-level Likert-type response scales (always, usually, sometimes, never), and all allowed respondents to indicate that they did not know the response (these were recoded to the median response). Sensitivity analyses were conducted to assess the impact of the recoding on the results.

**Table 1:** Patient approach and views toward healthcare communication (PAV-COM) Questions from the MCBS*

These next questions are about practices sometimes associated with receiving medical care. Please tell me if you always, usually, sometimes, or never do the following:	
PA9.	Read about health conditions in newspapers, magazines, or on the Internet.
PA10.	Read information about a new prescription, such as side effects and precautions.
PA11.	Bring with you to your doctor visits a list of questions or concerns you want to cover.
PA12.	Leave your doctor’s office feeling that all your concerns or questions have been fully answered.
PA14.	Make sure you understand the results of any medical test or procedure.
PA15.	Talk with your doctor or other medical person about your options if you need tests or follow-up care.
Thinking about your relationship with your doctor, please tell me if the following statements always, usually, sometimes, or never happen:	
PA16.	My doctor listens to what I have to say about my symptoms and concerns.
PA20.	My doctor explains things to me in terms that I can easily understand.
PA21.	I can call my doctor’s office to get medical advice when I need it.

*The two factors are *approach to interactions* (PA9, PA10, PA11, PA14, PA15) and *views about physician’s healthcare communication* (PA12, PA14, PA15, PA16, PA20, PA21).

### Data analysis

Database management was conducted in SAS 9.1, and statistical analyses were conducted in STATA MP 11.2.

#### Construct validity

First we used factor analysis to assess how well the individual survey items measure the construct of patient approach and views toward healthcare communication (construct validity). To determine whether the 9 items of interest could be combined into a scale, we evaluated unidimensionality using pairwise chi-squared tests, and examined the statistical significance of ordinal correlations using Spearman’s rho and Kendall’s tau. Because all of the survey items had ordinal responses, we estimated a polychoric correlation (covariance matrix) to examine the association between the variables. The polychoric estimation accounts for potential variation in distances between the levels and assumes that the latent constructs underlying the ordinal responses are normally distributed continuous variables. Each element of the polychoric matrix represents the correlation of a bivariate Gaussian distribution between two latent variables. Maximum likelihood was used to estimate the matrix. Based on simulation evidence, the two-step maximum likelihood estimation provides a close approximation of the underlying construct, particularly in large samples where cell frequencies are high [18,19].

Using the 2002 data, we conducted an exploratory principal factor analysis. Following the Kaiser criterion, we examined only factors with eigenvalues above 1. The resulting factors were then ordered by the proportion of the variance explained. The factors were rotated to better examine correlations among the items. We performed both Varimax orthogonal and Promax oblique rotations on the principal factors to assist with the interpretation and reproducibility of the item structure [20]. To test whether factors were consistent for linear scoring, we conducted a confirmatory factor analysis using the 2005 MCBS responses [21]. We did not use factor loadings from the 2002 exploratory principal factor analysis as constraints in the confirmatory factor analysis.

After confirming the principal factors, we constructed an additive score ranging from 9 to 36 using responses from the 9 items from 2002 and 2005 data. We affirmed the score’s unidimensionality by estimating item-to-rest correlations and evaluating the reliability of internal consistency with Cronbach’s alpha. To improve the interpretation, the score was linearly translated to a scale ranging from 0 to 100, which we called the Patient Approach and Views toward Healthcare COMmunication (PAV-COM) scale; higher PAV-COM scores represent greater patient preparation and better views toward healthcare communication. Distributional properties of the score (i.e., mean, median, variance, interquartile range, and skewness) were examined.

#### Predictive validity

To assess the predictive validity of the PAV-Com score (its ability to predict expected outcomes), we examined the relationship between mean PAV-COM scores and health-related behavior fulfillment. We expected that patients with higher PAV-COM scores would fulfill more health behaviors. Health-related behaviors were assessed by asking respondents whether they “currently smoked cigarettes, cigars, or pipe tobacco” and if they had “a flu shot last winter” or “a shot for pneumonia.” They also were questioned about “the most recent time their blood pressure was taken by a doctor or other health professional” and “the most recent time their blood cholesterol was checked.” Fulfillment of cancer screening tests in the past year was assessed by asking if respondents had a mammogram, pap smear, prostate specific antigen (PSA) test, and digital rectal examination since last year’s interview. All health-related behaviors were assessed in the fall survey. The associations between the PAV-COM score and behaviors were stratified by survey year and tested for significance using multivariate linear regression models adjusting for age, gender, and race/ethnicity.

## Results

### Patient characteristics

The analytical sample consisted of 15,165 adults aged 65 and older. Respondent characteristics from the 2002 and 2005 surveys were similar (Table 2). In both samples, there were more females than males, and the majority of the sample was white. Compared to the 2005 sample, a greater percentage of respondents reported receiving pneumonia vaccinations and having cholesterol levels checked in 2002. However, in 2005 a lower percentage of respondents reported receiving cancer screenings (mammograms, pap smears, and digital rectal examinations) in the past 12 months.

**Table 2:** Respondent characteristics and health-related behaviors by survey year

	**2002**	**2005**	
	**No. (%)**	**No. (%)**	**p-value**
Sample Size	7781 (100%)	7384 (100%)	
Respondent Characteristics			
Median age in years	76	76	0.142
Gender			
Male	3171 (41%)	3092 (42%)	0.161
Female	4610 (59%)	4292 (58%)	
Race/Ethnicity			
White, non-Hispanic	6775 (87%)	6475 (88%)	0.044
African American	683 (9%)	590 (8%)	
Hispanic	174 (2%)	144 (2%)	
Other	149 (2%)	175 (2%)	
Health-Related Behaviors			
Does not smoke	6970 (90%)	6693 (91%)	0.028
Flu shot last winter	5557 (72%)	4943 (67%)	<0.001
Pneumonia shot within lifetime	5545 (71%)	5513 (75%)	<0.001
Blood pressure checked within the last 12 months	7392 (95%)	7027 (95%)	0.852
Cholesterol checked within the last 12 months	6315 (85%)	6319 (87%)	0.001
Cancer screening behaviors within the last 12 months			
Mammogram	2124 (51%)	1845 (48%)	0.006
Pap smear test	1487 (34%)	1264 (31%)	0.001
Prostate specific antigen (PSA) test	1924 (71%)	1895 (72%)	0.545
Digital rectal examination	1415 (50%)	1256 (46%)	0.002

### Exploratory principal factor analysis

**Figure 1:**
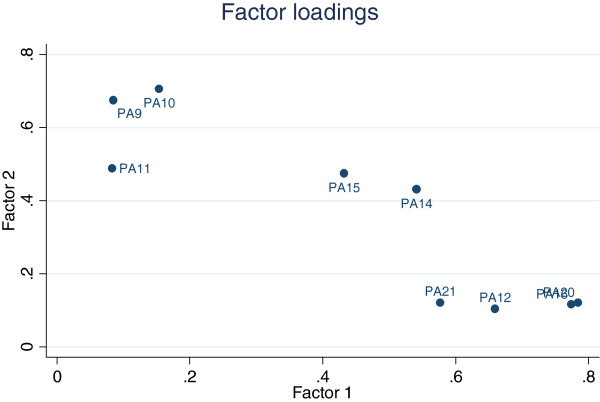
Factor loadings with varimax orthogonal rotation.

Unidimensionality tests indicated that the 9 items we examined on the MCBS surveys were related. Polychoric estimates ranged from 0.102 (PA11 and PA12) to 0.678 (PA16 and PA20). Principal factor analysis identified 2 factors with eigenvalues above 1 (Table 3). The first factor showed positive loadings for all items (0.337 to 0.722) and the second factor showed negative loadings on 4 items (PA12,PA16, PA20 and PA21). After orthogonal rotation, PA12, PA16, PA20 and PA21 separated from the other items (Figure 1). Among the remaining 5 items, 2 items (PA14 and PA15) contributed to both factors. We labeled the two factors *approach to interactions* (PA9, PA10, PA11, PA14, PA15) and *views about physician’s healthcare communication* (PA12, PA14, PA15, PA16, PA20, PA21). To assess effects of median recoding, the principal factor analysis was repeated after the removal of all “don’t know” respondents from the 2002 sample (6%), and yielded identical results.

**Table 3:** Factor loadings

	**Exploratory analysis, 2002**				**Confirmatory analysis, 2005**	
	**Unrotated**		Orthogonal*			
	**Factor 1**	**Factor 2**	**Factor 1**	**Factor 2**	**Factor 1**	**Factor 2**
PA9	0.441	0.518	0.085	0.675		0.672
PA10	0.515	0.506	0.154	0.706		0.774
PA11	0.337	0.363	0.083	0.488		0.558
PA12	0.609	−0.275	0.660	0.104	0.487	
PA14	0.689	0.064	0.541	0.431	0.343	0.244
PA15	0.622	0.161	0.432	0.475	0.337	0.313
PA16	0.711	−0.327	0.774	0.116	0.520	
PA20	0.722	−0.329	0.784	0.120	0.554	
PA21	0.549	−0.215	0.577	0.121	0.513	
Eigenvalue	3.1967	1.113				

* Loadings are illustrated in Figure 1.

### Confirmatory factor analysis

After conducting the exploratory factor analysis, which aids in the interpretation of a polychoric correlation matrix, we performed a confirmatory factor analysis using the 2005 data to confirm the association between the items and factors. The confirmatory factor analysis results confirmed the statistical significance of the factor loadings for each factor (ranging from 0.244 to 0.774), as well as the significance of the correlation between the 2 factors (0.261) (Table 3). In terms of conventional measures of fit, the root mean square error of approximation is 0.0763, the Tucker-Lewis index is 0.9037, and the comparative fit index is 0.9358.

### Patient Approach and Views about healthcare COMmunication (PAV-COM) scale

**Figure 2:**
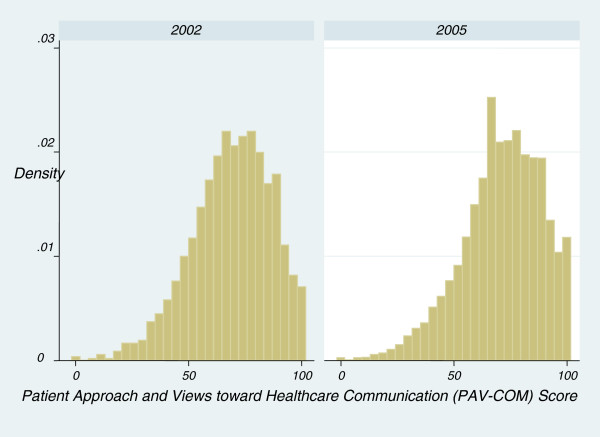
Histogram of Patient Approach and Views toward Healthcare Communication (PAV-COM) Score by Year.

The PAV-COM scale has good reliability, with a Cronbach’s alpha of 0.75. The subscales have Cronbach’s alpha of 0.76 (*approach to interactions)* and 0.69 (*views about physician’s healthcare communication*). Each item in the scale is positively correlated with a scale composed of the remaining items (correlations range from 0.33 to 0.53). In both 2002 and 2005, the distribution of the PAV-COM score was skewed slightly to the left (Figure 2). Between 2002 and 2005, the mean PAV-COM score increased from 68.9 to 71.2 (2.3; 95% CI 1.7, 2.9).

### Relationship between PAV-COM scores and health-related behaviors

Table 4 delineates adjusted differences in average PAV-COM scores by fulfillment of health-related behaviors. Overall, individuals who participated in cancer or health screenings and chose healthier behaviors had higher PAV-COM scores than those who did not. The smallest average difference in the PAV-COM score (2.7 points) was between respondents who were and were not screened using a Pap smear test (p < 0.001). The largest differences related to blood pressure being checked within the last 12 months.

**Table 4:** Differences in mean patient approach and views toward healthcare communication (PAV-COM) score by health-related behaviors and survey year and linearly adjusted for age, gender, race / ethnicity

	**Adjusted difference in mean PAV-COM score**					
	**2002**			**2005**		
**Health-related behavior**	**n**	**Difference**	**p-value**	**n**	**Difference**	**p-value**
Does not smoke	7781	4.68	< 0.001	7384	6.17	< 0.001
Flu shot last winter	7743	4.92	< 0.001	7361	4.36	< 0.001
Pneumonia shot within lifetime	7757	4.75	< 0.001	7373	5.25	< 0.001
Blood pressure checked within the last 12 months	7756	9.70	< 0.001	7378	8.86	< 0.001
Cholesterol checked within the last 12 months	7439	5.37	< 0.001	7274	5.37	< 0.001
Cancer screening behaviors within the last 12 months						
Mammogram	4197	4.82	< 0.001	3880	4.38	< 0.001
Pap smear test	4357	3.28	< 0.001	4110	2.68	< 0.001
Prostate specific antigen (PSA) test	2693	5.63	< 0.001	2625	7.11	< 0.001
Digital rectal examination	2812	3.54	< 0.001	2719	4.10	< 0.001

## Discussion

This study demonstrates that a 9-item scale to measure patient approach and views about healthcare communication with physicians has good internal consistency and validity. Two subscales were identified, *approach to interactions* and *views about physician’s healthcare communication*. To the best of our knowledge, this is the first study to examine the psychometric properties of a scale that measures both how actively patients approach encounters with healthcare providers, and how they view these interactions. Compared to patients with lower PAV-COM scores, those with higher scores more frequently obtained routine health screenings, cancer screenings, and vaccinations. These results are consistent with studies showing that patients who actively participate in medical encounters and have positive views about these interactions can influence processes of care [22]. However, further work should investigate whether patients received more services because they asked specifically for them when they might not otherwise have been offered, or whether increased preparation for office visits led patients to follow physician recommendations.

The effect of active patient preparation for visits on the physician-patient relationship is unknown. In one study, patients who were trained to be more involved in their medical care had better health outcomes [8,9], but they also may have experienced more anxiety and less satisfaction with their physicians than those not receiving the training [23]. There is some debate about whether patients who actively research health information have more productive interactions with healthcare providers [24] or whether their research breeds skepticism and mistrust of the medical system. Nonetheless, this study suggests that patients who more actively prepare for interactions with their providers and who have more positive views about communicating with their providers receive better preventive care.

The availability of nationally representative survey data presented a promising research opportunity; nevertheless, our study shares the survey’s limitations. It is uncertain whether the PAV-COM will produce similar results if different clinical relationships are examined, since survey questions asked patients to reflect on their relationship with a specific doctor. Further psychometric testing should be performed to ensure stability of the score in different patient subgroups and to assess its relationship with patient characteristics. Additional work also should be done to assess the concurrent validity of the scale.

Sampling weights for the older adult subsample of the MCBS do not account for non-response in the questions analyzed for this study, and would need to be re-estimated. Since this would contribute to parameter uncertainty, we did not apply weights for this estimation. Given the large sample size and the efforts of Centers for Medicare and Medicaid Services’ (CMS) to collect a nationally representative sample of Medicare beneficiaries, it is unlikely that the inclusion of study-specific sampling weights would noticeably improve the generalizability of the results.

The study also has other limitations. Active patient participation in healthcare is necessary to achieve patient-centered care. This measure represents limited aspects of patient-provider communication, since it does not assess actual patient expressions of concerns or feelings, or patient sharing of “health stories” in the context of everyday life [25]. This was a psychometric analysis of existing MCBS survey items, so some of the individual items might be construed to measure constructs other than communication. For example, the ability to call the physician’s office for advice when needed could be a measure of access to healthcare. Items such as reading about health conditions and about new medication prescriptions are generally considered to be health information seeking behaviors, rather than behaviors associated with preparing for interactions with a provider.

Preventive health measures were based on self-report, rather than on objective measures, such as medical records or claims data. Due to social desirability bias, patients who more actively sought healthcare information may have been more knowledgeable about desired preventive health behaviors and may have falsely reported fulfillment of the measures. Alternatively, these patients may have had greater awareness about whether they completed the health behaviors. However, even if the results merely reflect increased knowledge or awareness, it can be argued that patients who do not know about the recommended measures will be unable to complete them. Future research may examine the association between patient active communication and objective measures of preventive health behaviors.

## Conclusions

In conclusion, this study offers researchers a reliable and valid 9-item instrument containing 2 subscales (*approach to interactions* and *views about physician’s healthcare communication*)*,* for the assessment of patient approach and views toward healthcare communication. The causal relationship between the PAV-COM and the use of preventive health measures could be better assessed with a prospective study utilizing claims data or chart reviews to assess fulfillment of health behaviors.

## Competing interests

The authors declare that they have no competing interests.

## Authors’ contributions

All authors contributed to the concept, design, analysis and interpretation of the data, and were involved in drafting the manuscript. BMC conducted all study analyses. All authors read and approved the final manuscript.

## Pre-publication history

The pre-publication history for this paper can be accessed here:

http://www.biomedcentral.com/1472-6963/12/289/prepub
